# A Case of Immunoglobulin G4-Related Scleritis and Pneumonia Initially Diagnosed as Eosinophilic Pneumonia

**DOI:** 10.7759/cureus.29725

**Published:** 2022-09-29

**Authors:** Atsuki Fukushima, Hitoshi Tabuchi

**Affiliations:** 1 Ophthalmology, Tsukazaki Hospital, Himeji, JPN; 2 Ophthalmology, Hiroshima University, Hiroshima, JPN

**Keywords:** steroid, scleritis, pneumonia, igg4-related disease, eosinophil

## Abstract

Immunoglobulin G4 (IgG4)-positive plasma cells play a pivotal role in the pathogenesis of IgG4-related diseases, in which fibrosis is observed in various organs. Lesions often develop in the lacrimal gland and orbit; however, uveitis and scleritis are also noticed. We present the case of a 55-year-old woman who was diagnosed with eosinophilic pneumonia in November 2021 at the Department of Collagen Disease of another hospital. She was treated with 25 mg of oral prednisolone. On January 11, 2022, when the dose of prednisolone was reduced to 7.5 mg, she began complaining of conjunctival hyperemia in the left eye, and a few days later, eye pain was also reported. On January 17, she visited an ophthalmology clinic and was prescribed betamethasone and tacrolimus eye drops, and was subsequently admitted to our hospital. The blood test results showed a high IgG4 level. We consulted the collagen disease physician to evaluate her previous data. The serum IgG4 level collected on December 6, 2021, was 608 mg/dL, and a re-examination of the bronchial biopsy tissues on December 2, 2021, confirmed 48% of IgG4-positive cells. Thus, pulmonary lesions appeared to be IgG4-related pathologies. Increasing the oral prednisolone dose to 30 mg improved the scleritis. As seen in this case, the possibility of an IgG4-related disease should be considered when scleritis is observed in a patient diagnosed with eosinophilic pneumonia.

## Introduction

Immunoglobulin G4 (IgG4)-related diseases involve the infiltration of IgG4-positive plasma cells into various organs, including the pancreas causing organ damage due to fibrosis [1]. Lesions have been reported to occur more frequently in the lacrimal gland and orbit [2]. In Japan, IgG4-related diseases are reported as the second most common disease among orbital lymphoproliferative lesions [2]; however, they are not confined to the orbital region and may cause uveitis and scleritis [3].

Increased peripheral blood eosinophils and eosinophil infiltration in diseased tissues have been reported to be frequently observed in IgG4-related diseases [4]. Our previously reported case of IgG4-associated conjunctival mass was diagnosed and treated as eosinophilic sinusitis and eosinophilic pneumonia [5]. Although the present case was also diagnosed with eosinophilic pneumonia, IgG4-related disease was suspected when identifying the cause of scleritis, and the pulmonary lesion was found to be an IgG4-related disease. Thus, the possibility of an IgG4-related disease should be considered when scleritis is observed in a patient diagnosed with eosinophilic pneumonia.

## Case presentation

**Figure 1:**
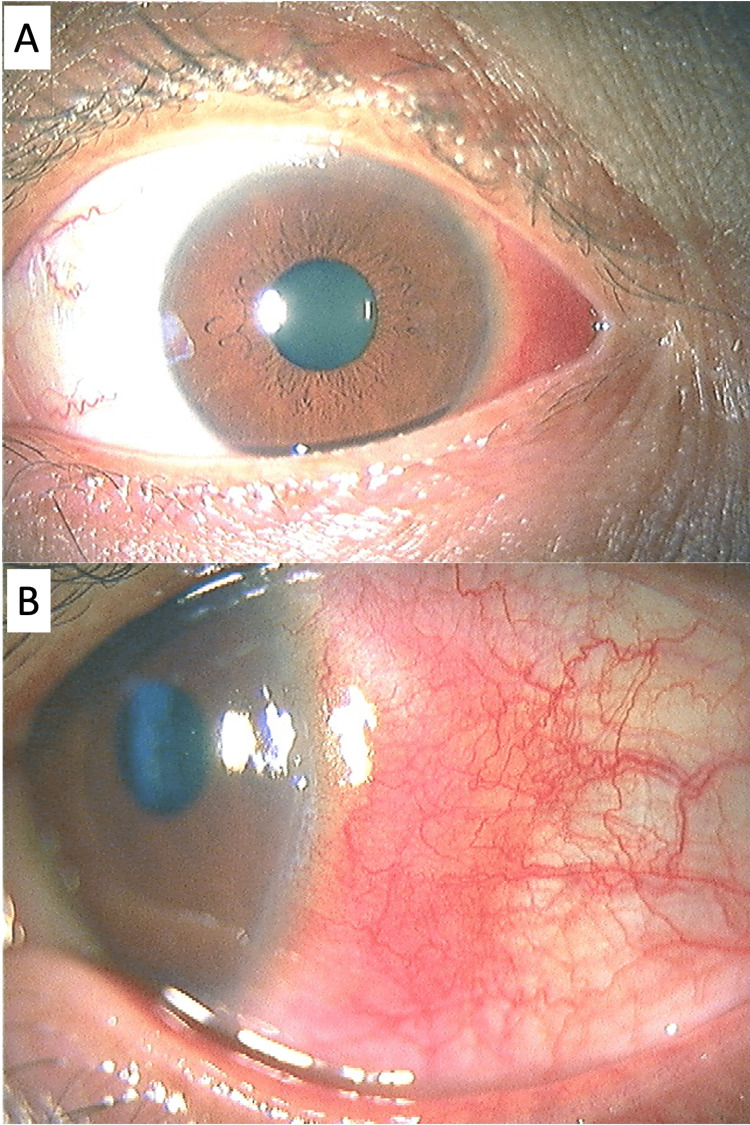
Slit photographs at the initial visit (March 2, 2022). (A) Lower magnification. (B) Higher magnification of the lateral side.

A 55-year-old woman was diagnosed with asthma and sinusitis in 2015. Further examination led to the diagnosis of eosinophilic pneumonia and she was treated with systemic administration of 25 mg of prednisolone since November 2021. On January 11, 2022, she started to complain of conjunctival hyperemia in her left eye, and a few days later, eye pain was noted. on January 17, she visited a nearby ophthalmology clinic and was prescribed 0.1% tacrolimus eye drops twice a day and 0.1% betamethasone eye drops four times a day for the left eye; however, no improvement or changes were observed. Therefore, she was referred to our hospital on March 2. At the first visit to our hospital, the dose of systemic prednisolone was reduced to 7.5 mg because her eosinophilic pneumonia was well controlled. Visual acuity was 0.8 (0.9) and 0.6 (1.2) in the right and left eyes, respectively, and the intraocular pressure was 8 mmHg in both eyes. There were no intraocular inflammatory findings; however, nodular scleritis was observed on the left temporal side (Figure 1).

**Figure 2:**
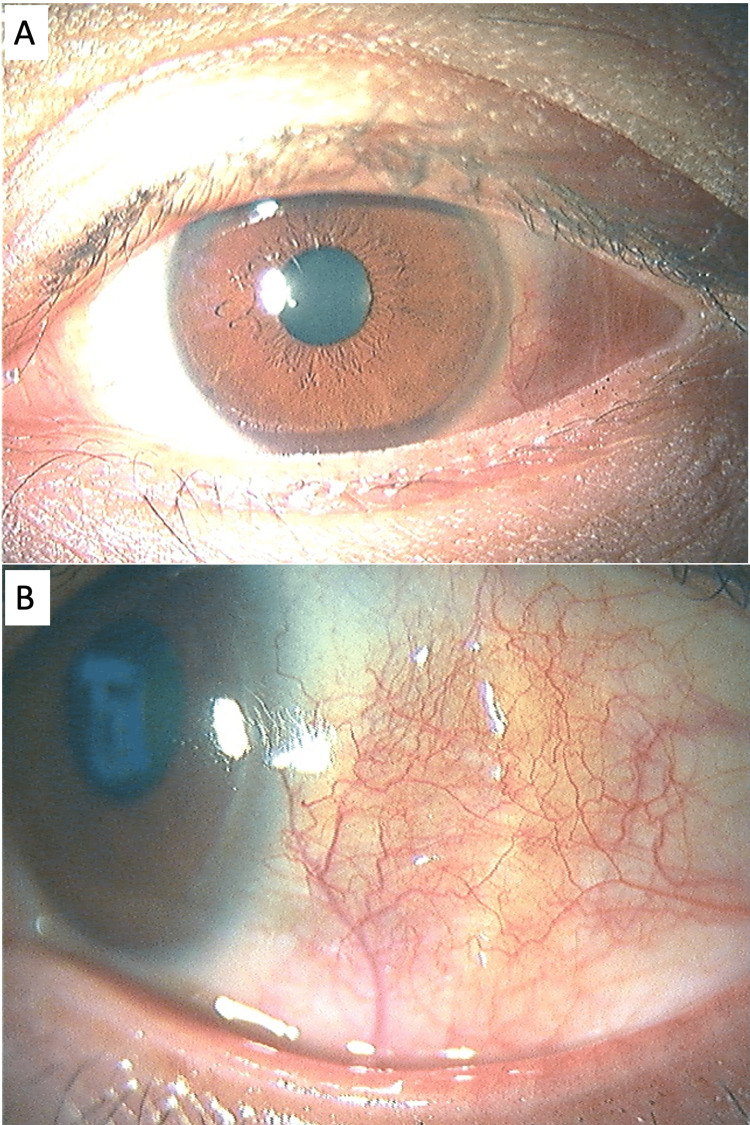
Slit photographs nine days after starting 30 mg of oral prednisolone (May 6, 2022). (A) Lower magnification. (B) Higher magnification of the lateral side.

The epiretinal membrane was observed in the right macular region, whereas no particular abnormality was observed in the left fundus. Subsequently, 0.1% tacrolimus eye drops were continued twice a day, and 0.1% betamethasone eye drops were increased to six times a day; however, her signs and symptoms did not improve. A blood test performed at the first visit demonstrated an elevated IgG4 level of 193 mg/dL (normal range: 11-121 mg/dL), and she was referred to the Department of Collagen Disease of another hospital where she was treated for eosinophilic pneumonia. When retested using the previously collected samples in the Department of Collagen Disease, the serum IgG4 level assessed on December 6, 2021, was 608 mg/dL, and the bronchial biopsy tissue on December 2, 2021, showed 48% IgG4-positive cells. Based on these findings, the collagen disease physician diagnosed the patient with IgG4-related lung disease instead of eosinophilic pneumonia (April 28, 2022). A 30 mg of prednisolone dosage was started orally on the same day. On May 15, 2022, computed tomography of the neck, chest, and abdomen was performed, which showed no abnormal findings, except for the remission of pulmonary lesions. Systemic administration of prednisolone improved conjunctival hyperemia and eye pain in the left eye (Figure 2).

**Figure 3:**
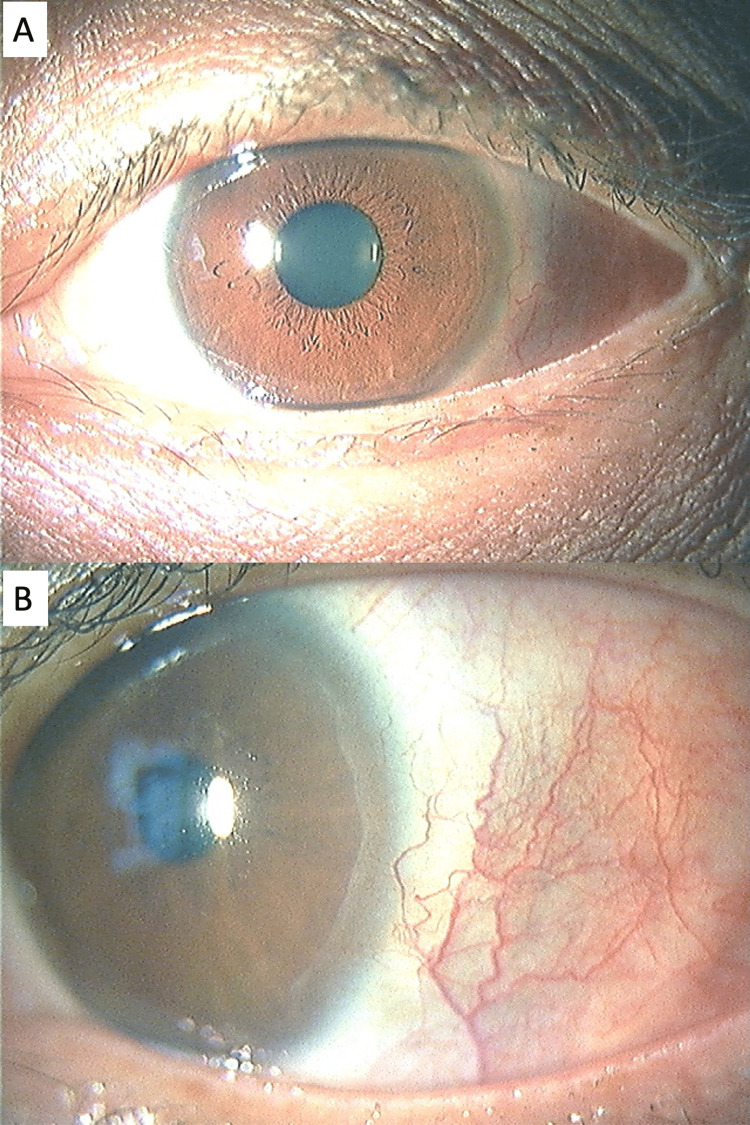
Slit photographs eight weeks after starting 30 mg of oral prednisolone (July 20, 2022). (A) Lower magnification. (B) Higher magnification of the lateral side.

She was given 0.1% tacrolimus eye drops twice a day, and 0.1% betamethasone four times a day was continuously administered. Her oral prednisolone dosage was reduced to 17.5 mg, and checkups were continued (Figure 3).

## Discussion

IgG4-related diseases are typically characterized by fibrotic and sclerotic changes observed in multiple organs due to the infiltration of lymphocytes and plasma cells [1]. In the field of ophthalmology, lacrimal gland inflammation and orbital masses have been reportedly associated with IgG4-related diseases [2]. Scleritis as an IgG4-related disease was first reported in 2012 [6]. In most cases, scleritis was found to be an IgG4-related disease after performing blood sampling for a detailed examination. Characteristically, it is often unilateral and does not involve other organs [7-14].

In the present case, a patient who was treated for eosinophilic pneumonia developed scleritis, and during follow-up, the pulmonary lesions were found to be an IgG4-related disease. Similarly, we reported a case of a conjunctival mass in a patient who was treated for eosinophilic pneumonia and eosinophilic sinusitis and was finally diagnosed to be an IgG4-related disease [4]. Thus, IgG4-related diseases should be considered even in patients diagnosed and treated with eosinophilic diseases in other departments.

Based on the results of previous prospective studies, the percentage of peripheral blood eosinophils increases by 9% and 38% in healthy subjects and in those with IgG4-related diseases, respectively [15]. Moreover, 86% of patients with IgG4-related diseases have eosinophil infiltration in tissues, such as the pancreas [15]. Although the role of eosinophils in IgG4-related diseases remains unclear, they have been reported to be involved in the survival and maintenance of plasma cells, involvement in T-cell infiltration and activation, and activation of macrophages and fibroblasts [4]. These reactions are consistent with the findings observed in the diseased tissues in IgG4-related diseases. Steroids are currently the main treatment; however, appropriate treatment methods for IgG4-related diseases should be developed to suppress the infiltration of eosinophils and plasma cells.

## Conclusions

When scleritis is present in patients who were diagnosed with eosinophilic pneumonia, IgG4-related disease should be considered because of the increased number of eosinophils in peripheral blood in patients with IgG4-related diseases.
